# Matrix model for collective phenomena in electron beam’s longitudinal phase space

**DOI:** 10.1038/s41598-021-87041-0

**Published:** 2021-04-12

**Authors:** Giovanni Perosa, Simone Di Mitri

**Affiliations:** 1 Dipartimento di Fisica, Università degli Studi di Trieste, Piazzale Europa 1, Trieste, Italy; 2 Elettra-Sincrotrone Trieste S.C.p.A., S.S. 14-km 163.5 in AREA Science Park, 34149 Basovizza Trieste, Italy

**Keywords:** Particle physics, Plasma physics

## Abstract

The possibility to predict, characterize and minimize the presence of spurious harmonic content in the longitudinal profile of high brightness electron beams, namely the microbunching instability, has become vital to ensure accurate modeling and reliable operation of radiofrequency and plasma-based linear accelerators such as those driving free-electron lasers. Recently, the impact of intrabeam scattering (IBS) on the instability has been experimentally demonstrated by the authors. This work complements that experimental study by extending existing theories in a self-consistent, piece-wise calculation of IBS in single pass linacs and multi-bend transfer lines. New expressions for the IBS are introduced in two different semi-analytical models of microbunching. The accuracy of the proposed models and the range of beam parameters to which they apply is discussed. The overall modeling turns out to be a fast comprehensive tool for the optimization of linac-driven free-electron lasers.

## Introduction

Modern science found its fortune in the disposal of high brightness charged particles and photon beams to advance its frontiers. In particular, electron beams have become crucial for several operations, ranging from X-ray free-electron lasers^1–4^ (FELs) and plasma accelerators^5–7^ to coherent electron cooling^8^ and generation of high power THz broadband radiation^9^. A common need of all these initiatives is preservation and control of the electron beam quality, commonly parametrized by brightness. For instance, matter exploration benefits from stable narrow spectral lines of FELs, granted by longitudinal coherence. However, to date its extension to the water window (from 300 to 500 eV) and higher photon energy results uncertain because of apparently partial understanding and not reproducible evidence of microbunching instability (MBI)^10–13^. As the name suggests, this phenomenon is a cascade process that enhances initial non-uniformities of the charge distribution. Because of its broadband gain, it is able to capture and amplify modulations from several $\upmu$m to few 100’s of $\upmu$m wavelength. Bunch length compression blueshifts these wavelengths by a factor 10–100, reaching the scale of the FEL cooperation length^14^. The large amplitude $\upmu$m-scale modulations in the final electron beam longitudinal phase space translate into large slice energy spread^15^, causing the reduction of photon brilliance. Moreover, the mixing of MBI-frequencies and FEL coherent emission generates shot-to-shot fluctuations of the multiline FEL, leading to the appearance of sidebands at specific frequencies^16,17^.

The instability is usually mitigated by Landau Damping^18^, phase mixing^19^ or a combination of both^20^. The first approach makes use of a laser heater (LH)^21^ in the low energy region of the linac; the second one is implemented via linear optics control through dispersive regions.

The standard analysis of MBI relies on the linearization of Vlasov–Maxwell equation for the electrons distribution $f(\vec {X})$^22,23^ passing through a magnetic bunch length compressor^24^. The 1-D bunching factor *b*, defined as the Fourier transform of the bunch longitudinal charge distribution,

1 $$\begin{aligned} b(k;s) = \frac{1}{N}\int d\vec {X} e^{-ikz}f(\vec {X}), \end{aligned}$$

is a broadband shot-noise-like spectral distribution. This is amplified by longitudinal space charge (LSC) along accelerating sections and coherent sinchrotron radiation (CSR) in dispersive regions. The effect of LSC and CSR on the formation of microbunches results in an integral equation with specific initial condition^23^:

2 $$\begin{aligned} b[k(s);s] = b_0[k(s);s] + \int _0^s d\tau K(\tau ,s)b[k(\tau );\tau ], \end{aligned}$$

Hereafter we call this ensemble of assumptions and expressions Huang–Kim model (HK). Together with density and energy modulations, the formalism allows one to determine the instability gain *G*, defined as the ratio between final and initial bunching factor. The dominant terms of the expression are

3 $$\begin{aligned}&G \approx \vert (1 - iR_{56}\Delta p(k_0;0))\vert e^{-\frac{1}{2}C^2k^2R_{56}^2\sigma _{\delta }^2}, \end{aligned}$$

and the effective energy spread induced by energy modulations^15^

4 $$\begin{aligned} \sigma _{E}^{(MBI)} = (mc^2)^2\frac{1}{2\pi n_z}\int dk |G(k)^2\Delta \gamma (k)^2|. \end{aligned}$$

$R_{56}$ is the element of the transfer matrix that couples the longitudinal coordinates $(z,\delta )$, $\Delta p(k_0;0)$ is an initial energy modulation and $\Delta \gamma (k)$ is the energy modulation in Lorentz unit per unit bunching factor, *C* is the bunch length compression factor implemented at a 4-dipoles magnetic chicane, *k* is the modulation wave number, $\sigma _{\delta }$ is the relative energy spread at the entrance of the compressor. The complete expression for the gain can be found in the reference^23^.

Similarly, the beam harmonic content can be obtained following the Bosch–Kleman (BK) formalism^25^. Differently from the HK model, whose results are derived for magnetic bunch length compressors only, the BK allows the description of MBI in linear regime along dispersive sections of arbitrary geometry, such as multi-bend transfer lines. The theory treats a 2-dimensional vector space of longitudinal modulations of electrons bunches. All the collective phenomena, namely LSC, CSR and coherent edge radiation (CER), possibly including the effect of vacuum chamber shielding, are introduced by means of impedances $Z(\lambda )$^26^, as a function of the modulation wavelength $\lambda$. The contribution to beam modulations are expressed via matrix multiplication. Although we limit our discussion to LSC, CSR and CER, it is always possible to add piece-wise other longitudinal collective effects, as long as they can be described by impedances. This makes the model highly adaptable to any kind of section and to any degree of accuracy in the description of the beam line.

Given the integrated impedance $Z_{col}(\lambda )$ of a specific collective effect along a section of length *L*, the corresponding matrix acting on the modulations space is

5 $$\begin{aligned} S_{L} = \frac{1}{E(L)} \begin{pmatrix} E(L) &{} 0 \\ -Z_{col}(\lambda )I &{} E(0) \end{pmatrix} \end{aligned},$$

where *E*(*z*) is the energy value along the section, with $0<z<L$.

When the section is energy-dispersive, the effect of longitudinal Landau Damping can be included through the following matrix

6 $$\begin{aligned} {\mathcal {D}} = \begin{pmatrix} F(\lambda ) &{} ik(\lambda )R_{56}CF(\lambda ) \\ iCG(\lambda )/E &{} CF(\lambda ) - k(\lambda )R_{56}C^2G(\lambda )/E \end{pmatrix} \end{aligned},$$

where $C = (1 - hR_{56})^{-1}$ and the functions *F* and *G* are

7 $$\begin{aligned} F(\lambda ) = \int \cos [k(\lambda )CR_{56}\delta /E]f(\delta )d\delta \quad \text {and} \quad G(\lambda ) = \int \sin [k(\lambda )CR_{56}\delta /E]f(\delta )\delta d\delta. \end{aligned}$$

These quantities embody the suppression of gain due to uncorrelated energy spread and they depend on the slice energy profile. Authors considered $f(\delta )$ as a normalized Gaussian function with rms energy spread $\sigma _{E}$. More precisely, the dynamics of microbunching inside a dispersive region is regulated by the harmonic content of the slice energy distribution, i.e. by its Fourier transform.

This paper concerns the theoretical description of intrabeam scattering (IBS)^27^ and its integration in a comprehensive MBI model. Recently, the accelerator community shows a renewed interest in IBS effect in linear accelerators and linac-driven X-ray FELs^28–31^. When the study involves a careful characterization of the electron beam longitudinal phase space, IBS cannot be considered as a minor effect. In a previous work^30^, we demonstrated experimentally the need of inclusion of IBS to correctly understand the development of MBI, especially at high gain values. To do so we used the HK formalism for MBI and a set of equations semi-analytically solved for the IBS. In this paper, we derive completely analytic expressions for the slice energy spread induced by IBS. These results are inserted in both HK and BK models, keeping track of the IBS-induced energy spread and updating piece-wisely the beam slice energy distribution. Starting with a specific but arbitrary profile for $f(\delta )$ and associated energy spread $\sigma _{E}(0)$, this value will be increased after a L-long section by the collisions between particles, resulting in an enlargement of the distribution width. To quantify $\sigma _{E}(L)$, or equivalently the relative uncorrelated energy spread $\sigma _{\delta }(L)$, we recall an expression for the energy spread growth rate and integrate it. In addition to this, the LH modeling is upgraded with respect to the 1-D approximation adopted in BK^25^. In order to consider the spatial superposition of laser and electrons^32^, we treat the whole 3-D configuration space, for arbitrary beam energy distributions.

## Results

### IBS growth rate

Historically, there are two consistent approaches to characterize the growth rate of transverse and longitudinal emittances. The first one, due to Piwinski^33^, is based on Analytical Mechanics; the second one, due to Bjorken and Mtingwa^34^, is based on Quantum Field Theory. These formalisms coincides in the high energy approximation^35^. In the framework of beam-driven light sources, we are particularly interested to the growth rate of the relative energy spread, whose differential equation is^35^:

8 $$\begin{aligned} \frac{1}{\tau _{\delta }} = \frac{1}{\sigma _{\delta }}\frac{d\sigma _{\delta }}{dt} = \frac{cr_e^2 N_e[\log {}]\sigma _H}{16\gamma ^2(\epsilon _{nx}\sigma _x)^{1/2}(\epsilon _{ny}\sigma _y)^{1/2}\sigma _z\sigma _{\delta }^3}g\left[ \bigg (\frac{\beta _x\epsilon _{n_y}}{\beta _y\epsilon _{n_x}} \bigg )^{1/2} \right], \end{aligned}$$

where *c* is the speed of light, $r_e$ is the classical electron radius, $N_e$ is the number of electrons, $\sigma _{\delta } = \sigma _{E}/E$, $\gamma = E/mc^2$, $\epsilon _n$ is the normalized emittance, $\sigma _{x,y}$ are the beam’s rms sizes, $\beta _{x,y}$ are the betatron functions, $\sigma _z$ is the bunch length,

9 $$\begin{aligned} \frac{1}{\sigma _H^2} = \frac{1}{\sigma _{\delta }^2} + \frac{\gamma {\mathcal {H}}_x}{\epsilon _{n_x}} + \frac{\gamma {\mathcal {H}}_y}{\epsilon _{n_y}}, \quad {\mathcal {H}}_{x,y} = \frac{\bigg [\eta _{x,y}^2 + \bigg (\beta _{x,y}\eta _{x,y}' - \frac{1}{2}\beta _{x,y}'\eta _{x,y}\bigg )^2\bigg ]}{\beta _{x,y}}, \end{aligned}$$

in which $\eta$ is the dispersion lattice function, and finally

10 $$\begin{aligned} g(x) = \frac{4\sqrt{x}}{\pi }\int _0^{\infty } \frac{y^2dy}{\sqrt{(1 + y^2)(x^2 + y^2)}}\left( \frac{1}{1 + y^2} + \frac{1}{x^2 + y^2} \right). \end{aligned}$$

Both the Piwinski^33^ and Bjorken-Mtingwa^34^ theory includes the so-called Coulomb logarithm:

11 $$\begin{aligned}{}[\log {}] = \ln {\left( \frac{b_{max}}{b_{min}}\right) } \approx \ln {\left( \frac{\theta _{max}}{\theta _{min}}\right) }, \end{aligned}$$

with $b_{max}$ and $b_{min}$ the maximum and minimum impact parameter of IBS scattering events, $\theta _{max}$ and $\theta _{min}$ the maximum and minimum scattering angle. Equation (11) inherits the logarithmic behaviour from the phase space divergence in the presence of long-range interactions; the divergence can be avoided by imposing a cutoff. This procedure is mandatory not only to normalize the integration in the phase space, but also to discard hard scattering events, which may heavily bias IBS contributions in the bunch core.

The minimum scattering angle is usually chosen in terms of Debye length or beam size. The lower limit is taken to be

12 $$\begin{aligned} \theta _{min} \approx \frac{2r_e m^2c^2}{b_{max}p^2} = \frac{2r_e }{\sigma _x\bar{\beta }^2}, \end{aligned}$$

choosing $b_{max} = \sigma _x$, (see^30^), and *p* is the momentum in the C.o.M. system. We propose a new estimate for the maximum scattering angle^30^, inspired to the strategy proposed for synchrotrons^36^. The upper limit is given in terms of the momentum transfer in the collision, *q*, calculated as the boundary beyond which the integrated scattering rate matches a characteristic time $\tau$. In the absence of equilibrium conditions, as they commonly happen to be in a storage ring, $\tau$ becomes here the time the beam takes to travel along the accelerator.

We start from^36^

13 $$\begin{aligned} q^2_{max} = \frac{c\tau N_er^2_e}{2\pi \gamma ^2\epsilon _x\epsilon _y\sigma _z\sigma _{\delta }}\int _0^{\infty } \frac{dx}{\sqrt{x^4 + ux^3 + vx^2 + wx}}, \end{aligned}$$

where the relation between *q* and $\theta$ is^33^

14 $$\begin{aligned} q = \frac{\bar{\beta }}{2}\sin {\bigg ( \frac{\theta }{2} \bigg )} \approx \bar{\beta }\theta , \end{aligned}$$

and $\bar{\beta }$ is the average normalized velocity in the C.o.M. system and the coefficients *u*, *v* and *w* are^36^

15 $$\begin{aligned}&u = \gamma ^2\bigg ( \frac{\gamma {\mathcal {H}}_{x}}{\epsilon _{n_x}} + \frac{1}{\sigma _{\delta }^2} + \frac{\beta _x}{\gamma \epsilon _{n_x}} + \frac{\beta _y}{\gamma \epsilon _{n_y}} \bigg ), \end{aligned}$$

16 $$\begin{aligned}&v = \gamma ^2\bigg ( \frac{\gamma ^2 {\mathcal {H}}_{x} \beta _y}{\epsilon _{n_x}\epsilon _{n_y}} + \frac{\beta _x\beta _y}{\epsilon _{n_x}\epsilon _{n_y}} + \frac{\gamma \beta _x}{\epsilon _{n_x}\sigma _{\delta }^2} + \frac{\gamma \beta _y}{\epsilon _{n_y}\sigma _{\delta }^2} + \frac{\gamma ^2\eta _{x}^2}{\epsilon _{n_x}}\bigg ), \end{aligned}$$

17 $$\begin{aligned}&w = \gamma ^2\bigg ( \frac{\gamma ^3\eta _{x}^2\beta _y}{\epsilon _{n_x}^2\epsilon _{n_y}} + \frac{\gamma ^2\beta _x\beta _y}{\epsilon _{n_x}\epsilon _{n_y}\sigma _{\delta }^2} \bigg ). \end{aligned}$$

For a round ($\epsilon _x = \epsilon _y$, $\beta _x = \beta _y$) and ultra-relativistic electron beam in a *straight* section, the polynomial’s coefficients reduce to:

18 $$\begin{aligned} u = \frac{\gamma ^2}{\sigma _{\delta }^2}, \qquad v = 2\frac{\beta _x}{\epsilon _n}\frac{\gamma ^3}{\sigma _{\delta }^2} = 2\frac{\beta _x\gamma }{\epsilon _n}u = 2\chi u, \qquad w = \frac{\beta _x^2}{\epsilon _n^2}\frac{\gamma ^4}{\sigma _{\delta }^2} = \chi ^2 u, \end{aligned}$$

and the integral can be rewritten in the simpler form

19 $$\begin{aligned} I = \int _0^{\infty } \frac{dx}{\sqrt{x}\sqrt{x^3 + u(x + \chi )^2}}. \end{aligned}$$

We now apply a change of variable of the form $x = \chi /(z-1)$, we get

20 $$\begin{aligned} I = \frac{1}{\chi }\frac{1}{\sqrt{\delta }} \int _1^{\infty } \frac{dz}{\sqrt{z^3 - z^2 + 1/\delta }}, \end{aligned}$$

where $\delta = u/\chi = \gamma \epsilon _n/\beta _x\sigma _{\delta }^2$. We note that in high brightness electron beams, and even for multi-GeV beam energies, $\delta$ is a small quantity, of the order of $10^5$–$10^6$. This allows us to take into account only terms of the order of ${\mathcal {O}}(1/\sqrt{\delta })$. We therefore rewrite Eq. (13) as:

21 $$\begin{aligned} q^2_{max} \approxeq \frac{c\tau N_e r_e^2}{2\gamma ^{3/2}\epsilon _n^{3/2}\sigma _z\sqrt{\beta _x}}. \end{aligned}$$

The mean value of the electron velocity in the C.o.M. frame is $\bar{\beta } = \frac{\gamma \sigma _{x'}}{\sqrt{2}}$^30^. This is substituted in the expression for the angles in (11), getting

22 $$\begin{aligned} \log {\bigg (\frac{\theta _{max}}{\theta _{min}}\bigg )} = \log {\bigg (\frac{q_{max}\sigma _x\bar{\beta }}{2r_e}\bigg )} = \log {\bigg (\frac{q_{max}\epsilon _n}{2\sqrt{2}r_e}\bigg )}. \end{aligned}$$

Differently from our first extimation^30^, we can conclude that, at first order in $\delta$, the Coulomb logarithm does not depend on $\sigma _{\delta }$, but it does depend upon $\gamma$. The implication of our new findings compared to our first estimates are illustrated and discussed below.

For an ultra-relativistic beam in a *dispersive* section, instead^36^,

23 $$\begin{aligned} u = \frac{{\gamma }^{2}}{\sigma _H^2}, \qquad v = \frac{\gamma ^2\beta _y}{\sigma _H^2\epsilon _{n_y}} + \frac{\gamma ^2\tilde{\sigma }_x^2}{\sigma ^2\epsilon _{n_x}^2} = \alpha u + \zeta , \qquad w = \frac{\gamma ^2\beta _y\tilde{\sigma }_x^2}{\sigma ^2\epsilon _{n_x}^2\epsilon _{n_y}} = \alpha \zeta . \end{aligned}$$

Again, the integral can be rewritten in the simpler form

24 $$\begin{aligned} I = \int _0^{\infty } \frac{dx}{\sqrt{x}\sqrt{x^3 + (ux + \beta )(x + \alpha )}}. \end{aligned}$$

Since $\alpha u /\zeta \approx 1$ and defining the coefficient $\omega = \zeta /\alpha ^2$, we obtain

25 $$\begin{aligned} \frac{1}{\alpha }\int _0^{\infty } \frac{dy}{\sqrt{1 + \omega y(y + 1)^2}} = \frac{1}{\alpha \sqrt{\omega }}\int _1^{\infty } \frac{dz}{\sqrt{1/\omega + z^3 - z^2}} \propto \frac{1}{\alpha \sqrt{\omega }} = \frac{1}{\sqrt{\alpha u}}. \end{aligned}$$

Notice that $\alpha$ plays the role of $\chi$ in the non-dispersive case, but now *u* depends on $\sigma _H$. As a consequence, the Coulomb logarithm has the same formal expression of the non-dispersive case, but now $q_{max}$ is the expression (13) times the ratio $\sigma _{H}/\sigma _{\delta }$.

Again, it is important to underline that these integrals at first order in $\delta$ are analytically exact, differently from the numerical one used so far^30^. On top of the higher precision reached in the determination of the Coulomb logarithm, which was still ambiguous in dispersive regions, its functional dependence from the beam energy and from the energy spread is now explicit, leading to new expressions for the induced energy spread.

### IBS-induced energy spread

With the proposed expression for the Coulomb logarithm in Eq. (22), we can proceed and solve Eq. (8). We discriminate two cases: dispersive section at constant energy and straight (or non-dispersive) section, in the presence or absence of acceleration. In the latter configuration, the beam energy is assumed to grow linearly along the section with a null dispersion (${\mathcal {H}}_{x,y} = 0$) and a gradient $G = [E(L) - E(0)]/L$, i.e.

26 $$\begin{aligned} d\gamma = \frac{G}{mc^2}cdt = \frac{G}{mc^2}ds. \end{aligned}$$

#### Straight section with varying energy

For the moment, we simplify the math by assuming a round beam. Thus, the function in (10) becomes $g(1) = 2$ and the growth rate differential equation is simplified:

27 $$\begin{aligned} \frac{1}{\tau _{\delta }} = \frac{cr_e^2N_e[\log {}](\gamma )}{8\gamma ^2\epsilon _n\sigma _x(\gamma )\sigma _z\sigma _{\delta }^2}, \end{aligned}$$

where we make explicit the dependence from $\gamma$ of the terms. Eq. (8) can be recast in the following form

28 $$\begin{aligned} \frac{d \sigma _{\delta }^2}{d\gamma } = \frac{r_e N_e mc^2 [\log {}](\gamma )}{4G\gamma ^2\epsilon _n\sigma _x(\gamma )\sigma _z} = k\frac{[\log {}](\gamma )}{\gamma ^{3/2}} \quad \text {with} \quad k = \frac{r_e N_e mc^2}{4G\epsilon _n^{3/2}\beta _x^{1/2}\sigma _z}. \end{aligned}$$

Using the Fourier Method we get

29 $$\begin{aligned} \sigma _{\delta }^2(\gamma ) - \sigma _{\delta }^2(\gamma _0) = 3k\log {(q)}\bigg ( \frac{1}{\sqrt{\gamma }} - \frac{1}{\sqrt{\gamma _0}} \bigg ) - 2k\bigg ( \frac{\ln {(q/\gamma ^{3/4})}}{\sqrt{\gamma }} - \frac{\ln {(q/\gamma _0^{3/4})}}{\sqrt{\gamma _0}}\bigg ), \end{aligned}$$

where *q* is the part of the argument of the Coulomb logarithm which does not depend on $\gamma$.

#### Straight section with constant energy

In this case the separation of variables is immediate, leading to

30 $$\begin{aligned} \sigma _{\delta }^2(s) - \sigma _{\delta }^2(0) = \frac{r_e N_e [\log {}] s}{4\gamma ^{3/2}\epsilon _n^{3/2}\beta _x^{1/2}\sigma _z}. \end{aligned}$$

This expression can also be derived by looking at certain limits of the previous solutions. In fact, this results coincides with the first case, if $d\gamma$ tend to zero, since the ratio $d\gamma /G$ reduces to $L/mc^2$.

#### Dispersive section

The differential equation to be solved has the form:

31 $$\begin{aligned} \frac{d\sigma _{\delta }^2}{ds} = \frac{a\log {\big (b \frac{\sigma _{H}}{\sigma _{\delta }}\big )}}{\sqrt{h\sigma _{\delta }^2+1}} \quad \text {with} \quad a = \frac{r_e^2N_e}{4\gamma ^2\epsilon _n \sigma _x\sigma _z}, \quad b = \sqrt{c\tau a}\frac{\epsilon _n}{2r_e}, \quad h = \frac{\gamma {\mathcal {H}}_x}{\epsilon _n}. \end{aligned}$$

The equation can be integrated separating the variables,

32 $$\begin{aligned} \int \frac{\sqrt{h\sigma _{\delta }^2 + 1}d\sigma _{\delta }^2}{\log {\bigg ( \frac{b}{\sqrt{h\sigma _{\delta }^2 + 1}} \bigg )}} = \frac{2b^3}{h}\bigg \{Ei\bigg [3\log {\bigg (\frac{\sqrt{h \sigma _{\delta }(0) + 1}}{b}\bigg )}\bigg ] - Ei\bigg [3\log {\bigg (\frac{\sqrt{h \sigma _{\delta }(s) + 1}}{b}\bigg )}\bigg ]\bigg \} = as, \end{aligned}$$

in which *Ei* stands for the exponential integral^37^.

In order to find the value of $\sigma _{\delta }(s)^2$, we need to find numerically the zero of the function *F*, written as

33 $$\begin{aligned} F(x) = \frac{2b^3}{h}\bigg \{Ei\bigg [3\log {\bigg (\frac{\sqrt{h x_0 + 1}}{b}\bigg )}\bigg ] - Ei\bigg [3\log {\bigg (\frac{\sqrt{h x + 1}}{b}\bigg )}\bigg ]\bigg \} - as, \end{aligned}$$

If we take the limit for *h* that tends to zero, Eq. (33) coincide with (30).

### Laser heater

As indicated by Eqs. (3) and (7), the MBI can be damped by a large uncorrelated energy spread. The effect, however, depends on the specific beam energy distribution. Such energy Landau damping is in most cases obtained via the LH. The functionality of a LH has been succesfully tested already at several FEL facilities. Its positive effects on both coherent optical transition radiation and FEL performances are widely known^32,38,39^.

LH consists of an undulator placed at the center of a chicane in which the electron beam interacts with an external laser. The ongoing process is the formation of modulations at the laser wavelength. The chicane’s dispersion is arranged to smear the phase space structure, leaving the beam with an higher uncorrelated energy spread.

Following the standard procedure to characterize the interaction of the laser with the electron bunch, the process can be described as a shaping of the electron energy distribution^32^.

34 $$\begin{aligned} \rho (s,\Delta \gamma ,r) = f_l(s,\Delta \gamma )\times f_t(r) = \frac{I}{ec\sqrt{2\pi }\sigma _{\gamma }}\exp {\bigg \{-\frac{[\Delta \gamma - \Delta \gamma _{LH}(r)\sin {k_{LH}s}]}{2\sigma _{\gamma }}\bigg \}}\times \frac{1}{2\pi \sigma _x^2}\exp {\bigg (-\frac{r^2}{2\sigma _x^2}\bigg )}, \end{aligned}$$

where $\Delta \gamma _{LH}(r)$ is the amplitude of the modulation induced by the resonant interaction^32^

35 $$\begin{aligned} \Delta \gamma _{LH}(r) = \sqrt{\frac{P_L}{P_0}}\frac{KL_u}{\gamma \sigma _r}\bigg [ J_0\bigg (\frac{K^2}{4 + 2K^2}\bigg ) - J_1\bigg (\frac{K^2}{4 + 2K^2}\bigg ) \bigg ]\exp {\bigg (-\frac{r^2}{4\sigma _r^2} \bigg )}. \end{aligned}$$

$\Delta \gamma = \Delta \gamma _{LH}(0)$ and, assuming a round beam, we’re using cylindrical coordinates to describe the transverse part of the distribution. To obtain the energy distribution, it is sufficient to integrate $\rho$ over the spatial coordinates

36 $$\begin{aligned} f(\Delta \gamma ) = \int \rho (s,\Delta \gamma ,r)dV = \frac{1}{\pi \sigma _x^2\sqrt{2\pi }\sigma _{\gamma }} \int rdr\exp {\bigg (-\frac{r^2}{2\sigma _x^2}\bigg )}\int \frac{d\xi }{\sqrt{\Delta \gamma _{LH}(r) - (\Delta \gamma - \xi )^2}}\exp {\bigg (-\frac{\xi ^2}{2\sigma _{\gamma }^2}\bigg )}. \end{aligned}$$

In the original BK formalism, a simpler expression for F and G is given^25^ and the dependence of $\rho$ from the transverse coordinates is neglected. Here, we derive fully three-dimensional expressions for Landau damping terms, plugging $f(\Delta \gamma )$ in Eq. (7), to obtain

37 $$\begin{aligned} F_{LH}= & {} \exp {\bigg (-\frac{1}{2}k(\lambda )CR_{56}\sigma _{\delta } \bigg )}\int RdR\exp {\bigg ( - \frac{R^2}{2} \bigg )}J_0\bigg [k(\lambda )R_{56}\delta _{LH}\exp {\bigg ( -\frac{R^2\sigma _x^2}{4\sigma _r^2} \bigg )} \bigg ], \end{aligned}$$

38 $$\begin{aligned} G_{LH}= & {} \exp {\bigg (-\frac{1}{2}k(\lambda )CR_{56}\sigma _{\delta } \bigg )}\int RdR\exp {\bigg ( - \frac{R^2}{2} \bigg )}J_1\bigg [k(\lambda )R_{56}\delta _{LH}\exp {\bigg ( -\frac{R^2\sigma _x^2}{4\sigma _r^2} \bigg )} \bigg ]. \end{aligned}$$

These expressions allow to model the superposition of laser and electrons in the BK model and take into account the finite sizes of both laser spot and bunch transverse dimensions together with their eventual mismatch.

### Simulations

As a case of study, HK and BK models are implemented to study the microbunching instability gain at the FERMI^40^ linear accelerator. We use broadband shot-noise density modulation as initial condition for $b_0(\lambda )$ and compute the bunching evolution along each section of the beam line. In dispersive regions, for the first formalism we apply equation (3), while for the second we build the Landau damping matrix (7). Parameters used for each section are shown in Table 1. The FERMI linac is made of a photo-injector followed by two magnetic compressors (chicanes), and interleaved by accelerating sections. The model starts being applied to the beam at the injector exit, at the beam energy of approximately 100 MeV.

**Table 1:** Table of parameters used for the simulations of FERMI linac.

Parameters	Value	Units
Bunch charge	650	pC
Initial current	55	A
Initial beam energy	96	MeV
Beam energy at BC1	278	MeV
Beam energy at BC2	610	MeV
Final beam energy	894	MeV
$R_{56}$ of BC1	42	mm
$R_{56}$ of BC2	0	mm
Compression factor	11	
Normalized emittance	0.7–0.9	mm mrad
$\langle \beta _{x,y} \rangle$ along the linac	8–35	m

**Figure 1:**
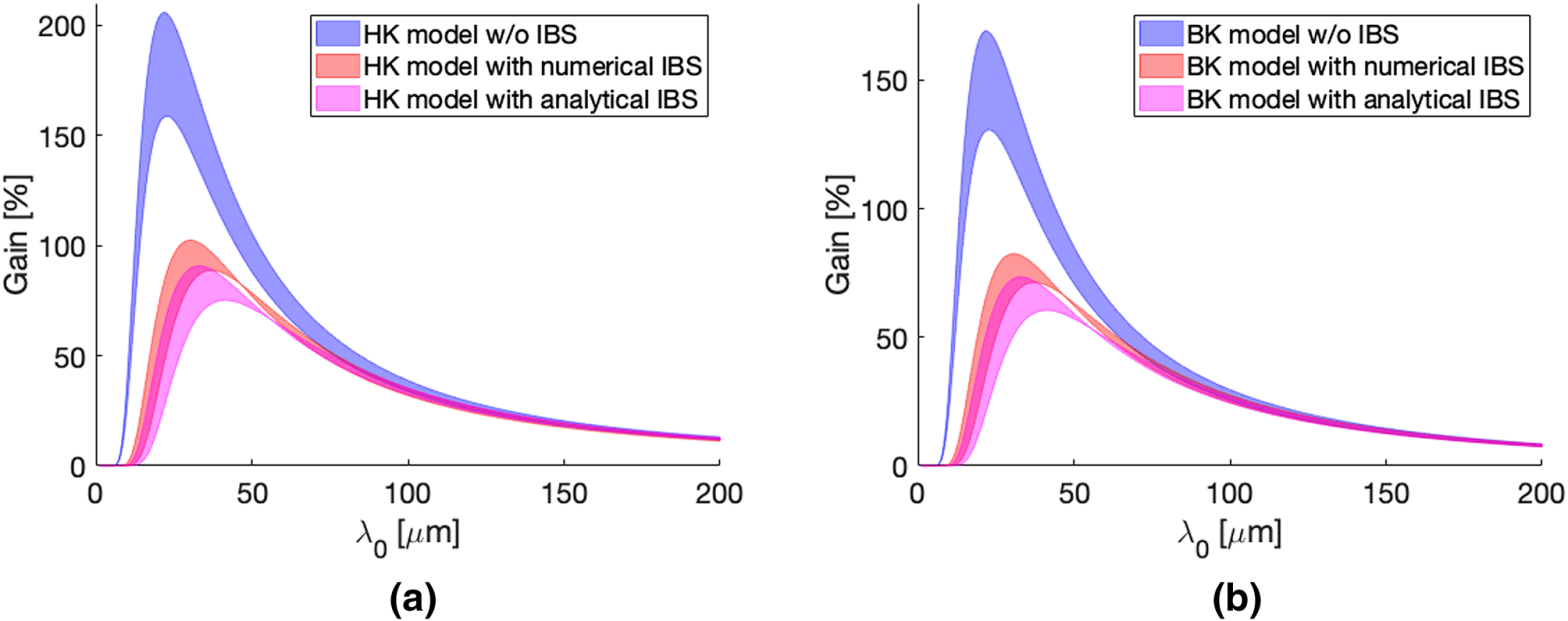
Microbunching gain for (**a**) Huang–Kim model and (**b**) Bosch–Kleman model. Blue lines correspond to MBI model without IBS, red lines to MBI with numerical IBS and magenta lines to MBI model with the newly introduced analytical expressions for IBS.

Figure 1 shows the spectral gain obtained for HK and BK model, without the introduction of IBS (blue lines), with IBS given by the approximated Coulomb logarithm^30^ (red lines) and with the new formulas (magenta lines), as in Eqs. (29), (30) and (33). Since our script uses mean values for $\beta$ functions and it is not able to reproduce possible strongly non-periodic behaviour along an accelerator section, we used shaded areas to take into account uncertainties on the optics modelling. Taking into account variation of the $\beta$s respect to their mean values up to 30% or so we observe a variation of $\sim 25\%$ of the gain peak in both HK and BK formalisms. This variation corresponds to an errorbar for $\sigma _{E}^{(MBI)}$ at the end of the linac of approximately $\pm \,30$ keV with the parameters in Table 1.

The overlapping region between the gain curves obtained with different IBS approaches, namely the numerical one^30^ and the one presented here, demonstrates the overall consistency of the two approaches. Still, the calculation of the Coulomb logarithm with the new expression tends to provide slightly larger growth rates of the energy spread and instability gain reduction. The comparison between plots in Fig. 1 underlines also a good agreement between the two models for the range of parameters considered here, and, in particular, a comparable reduction under the new IBS addition.

Bosch–Kleman formalism and the new expressions for IBS are also implemented to characterize microbunching instability at the end of the multi-bend transfer line (spreader) of FERMI. To authors’ best knowledge, this is the only way to describe in a semi-analytical manner the contribution of IBS to MBI in dispersive regions that are not chicanes. Parameters used for the linac are shown in Table 1, for the spreader in Table 2.

**Table 2:** Table of parameters used for FERMI transfer line.

Parameter	Value	Unit
Bunch current	600	A
Beam energy	894	MeV
$R_{56}$ of first dipole	0.18	mm
$R_{56}$ of second dipole	− 1.7	mm
$R_{56}$ of third dipole	− 1.7	mm
$R_{56}$ of fourth dipole	0.18	mm
$\langle \beta _{x,y} \rangle$ along the beam line	10–25	m

**Figure 2:**
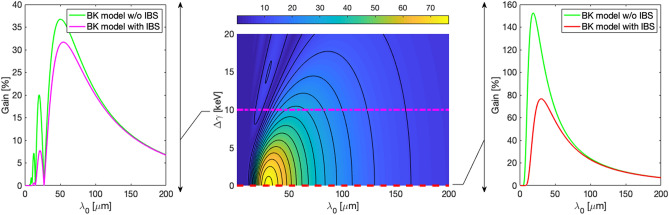
Center: Gain map obtained with BK formalism at the end of FERMI linac, as a function of modulation uncompressed wavelength and laser heater induced energy modulation. On the right, spectral gain profile with no LH. On the left, gain profile for a LH-induced energy modulation of 10 keV. Both profiles are also shown without IBS.

**Figure 3:**
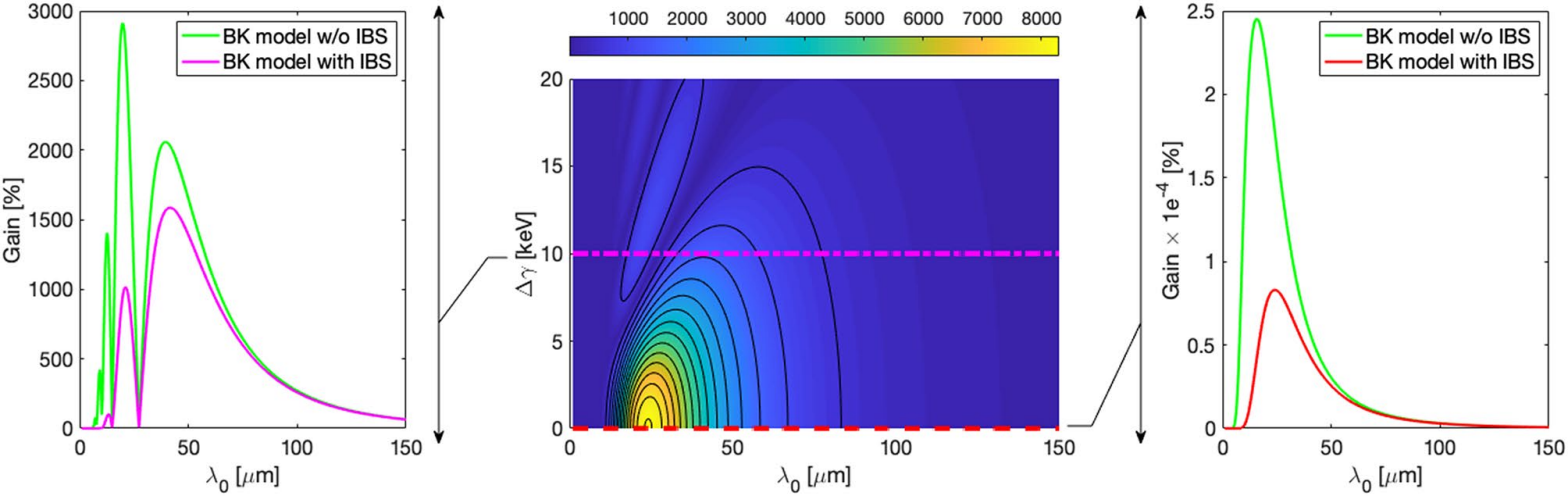
Same as in Fig. 2, but now the gain calculation is at the end of the FERMI spreader.

The gain is shown in Figs. 2 and 3 as a function of modulations uncompressed wavelength and of the energy modulation induced by the laser heater (using expressions (37) and (38)) at the end of the linac and at the end of the transfer line respectively. From these maps, it is possible to extract gain curves at different values of laser heater induced energy modulation. In particular, we show the results in two cases: absence of laser heater and 10 keV of induced energy modulation. Clearly, due to the presence of a long dispersive region, the gain blows up, but it is still kept under control by the effect of IBS, even in the presence of laser heater.

## Discussion

We discuss here the assumptions of our expressions. The proposed energy spread growth rate (29), (30) and (33), requires two conditions: ultra-relativistic energies and roundness of the transverse distribution. The first of this requirement is clearly fulfilled in each simulated section and can be used whenever the electrons kinetic energy is much greater than $m_e c^2$. The second, in general, is not always true. What is true, indeed, is that Twiss parameters in x and y tend to be of comparable magnitude when averaged over tens of meters long sections, but not locally equal. It is easy to show that our approximation leads to an overestimation of IBS effect: the function *g*(*x*) has a maximum when its argument is equal to 1. The eventual overestimated contribution, however, lies inside the error-bar due to the optics uncertainties.

The numerical and analytical treatments of Coulomb logarithm gives comparable results in the range of parameters considered here, and typical at short wavelength FELs. The consistency of the two approaches and the fact that the numerical one has been already benchmarked experimentally^30^ prove the validity of our formulas. Still, it is worth reminding that in the numerical strategy the integration of Eq. (8) is done discarding the functional dependence of the Coulomb logarithm from $\gamma$ and $\sigma _{\delta }$. The similarity between the two treatments, in the range of parameter of Table 1, can be interpreted considering that, at first order in $\delta$, the dependence of $[\log {}]$ from the energy spread disappears along straight sections, see Eq. (21). In spite of this, we believe that, in general, the analytical method is preferable to the other one: while the numerical approach uses a mean value for $\gamma$ and ignores its variation, our model keeps track of $\gamma (s)$ and is capable of more accurate predictions.

As for the assumption of linear gain of the instability, we admit that our model is not able to uniquely identify a gain threshold over which the linear regime is not valid anymore. Nonetheless, we can demonstrate that, for the range of parameters considered here, the presence of IBS guarantees the assumption to be valid. This fact is proved by the omnipresent factor 2 (or even higher) of gain reduction in our new model and, at the same time, by the consistency of the analytical results with experimental data presented elsewhere^30^. This analysis demonstrates once again that a proper inclusion of IBS in the MBI modelling is crucial to a faithfull description of MBI in linear regime.

As a final point, we discuss under which circumstances the growth of uncorrelated energy spread due to IBS can be neglected. Since the energy distribution and the bunching factor are updated step-wisely, by discretizing the curvilinear coordinate along the simulated beamline, the BK model is not able to fully couple the dynamics of MBI, as instead permitted by massive numerical calculations by Vlasov solvers^22,31^. To give a quantitative answer, we look at the microbunching gain as defined in HK model, Eq. (3), and plug in the analytically derived IBS terms (see Supplementary Material. Doing so, we derive explicit conditions under which the collisions can be neglected. To this purpose, it is easier to simplify Eq. (32), assuming that the uncorrelated energy spread remains small and keeping only the linear terms in *s*, obtaining

39 $$\begin{aligned} \sigma _{\delta }(s) \approx \sigma _{\delta }(0) + a\log {(b)}s =\sigma _{\delta }(0) + A s. \end{aligned}$$

Considering this formula in the derivation of the gain, following the strategy and the assumptions of HK model^23^, we find that IBS term can be neglected for

40 $$\begin{aligned} \lambda \gg \lambda _{crit} = \frac{\pi L_b C^2 R_{56}^2 A}{2\sqrt{\epsilon \beta }\nu }, \end{aligned}$$

where $\nu$ is the chicane bending angle.

For the parameters in Table 1, $\lambda _{crit} \approx 2\times 10^{-9}$ m and since the FEL process is affected by modulations at the micron scale, we are legitimized to neglect all the contributions. This result justifies the partial decoupling of MBI and IBS in our model.

## Conclusion

The contribution of intrabeam scattering, taking into account the approximated functional dependence of the Coulomb logarithm from beam energy and energy spread, in arbitrary dispersive, non-dispersive and accelerating sections was analytically derived. We present here a new derivation of the expression for the maximum value of the momentum transfer in collisions, written as a function of the electron beam parameters. The expressions have been integrated into two different semi-analytical models for microbunching instability in linear regime. We have compared numerical and analytical results for IBS, finding a slightly stronger damping of the instability by IBS in the analytical model, but still compatible with experimental data presented in literature^30^. This envisages the need for more accurate measurements of slice energy spread along the accelerator, possibly as function of varying beam parameters, of more accurate characterization of Twiss parameters along the beam line and the development of an even more realistic 3-D modelling of the instability based on the local optics values. Our analytical model results in a more accurate description of the momentum-space cutoff with respect to the numerical one, since it keeps track of the dependence of $q_{max}$ from $\gamma$. The approximations adopted in our modelling are all justified, so that the proposed analytical framework for the MBI in the presence of IBS allows to rapidly characterize MBI in a wide range of accelerator parameters, given the capability of Bosch-Kleman formalism to include several collective phenomena, knowing the associated impedances, and arbitrary multi-bend lines.

## Supplementary information

Supplementary Information.
