# Supplementary material for: Matrix model for collective phenomena in electron beam’s longitudinal phase space
Source: Sci Rep. 2021 Apr 12;11:7895. doi: 10.1038/s41598-021-87041-0 (PMC8041831; doi:10.1038/s41598-021-87041-0)
Supplement: Supplementary file 1 — Supplementary Information. [file 41598_2021_87041_MOESM1_ESM.pdf]

Supplementary material for
Matrix model for collective phenomena in electron beam's longitudinal phase space

Giovanni Perosa[1,*] **and Simone Di Mitri**[1,2]

[1]Università degli Studi di Trieste, Dipartimento di Fisica, Piazzale Europa 1, Trieste, Italy.
[2]Elettra-Sincrotrone Trieste S.C.p.A., S.S. 14-km 163.5 in AREA Science Park 34149 Basovizza, Trieste, Italy.
[*]giovanni.perosa@elettra.eu

Result

In this document, we derive the conditions under which energy spread growth due to intrabeam scattering can be neglected when considering the evolution of microbunching instability in a magnetic bunch length compressor. In doing so, we will consider the effect of IBS on the zeroth order term of the energy distribution $f(\vec{X};s)$. According to a recent numerical analysis[1], we can discard higher order effects on microbunching instability (MBI). We will follow the formalism and the notation proposed by Huang and Kim[2].
We start from the bunching factor $b[k(s);s]$, defined as

$$b[k(s);s] = \frac{1}{N}\int d\vec{X} e^{-ikz} f(\vec{X};s), \tag{1}$$

whose evolution is described by the integral equation

$$b[k(s);s] = b_0[k(s);s] + \int_0^s d\tau K(\tau,s) b[k(\tau);\tau], \tag{2}$$

where $b_0[k(s);s]$ is the bunching factor of the initial distribution $f_0(\vec{X}_0)$. The latter is decomposed in the following way:

$$f_0(\vec{X}_0) = \bar{f}_0(\vec{X}_0) + \hat{f}_0(\vec{X}_0), \tag{3}$$

in which the first term is the average distribution, assumed Gaussian in transverse and energy variables and uniform in z; the second term is an arbitrary but small perturbation. As anticipated, IBS acts increasing the width of the energy Gaussian.
The kernel of the equation is

$$\begin{aligned} K(\tau,s) = ik(s)R_{56}(\tau\to s)\frac{I(\tau)Z[k(\tau);\tau]}{\gamma I_A} e^{-k_0^2 U^2(s,\tau)\sigma_\delta^2/2}\times \\ \exp\Big[-\frac{k^2(s)\epsilon_0\beta_0}{2}\Big(V(s,\tau)-\frac{\alpha_0}{\beta_0}W(s,\tau)\Big)^2 - \frac{k^2(s)\epsilon_0}{2\beta_0}W^2(s,\tau)\Big] \end{aligned} \tag{4}$$

where k_0 is the modulation wave number at $s=0$, $k(\tau) = B(\tau)k_0$, $B(\tau) = [1+hR_{56}(s)]^{-1}$ is the compression factor, $I(\tau) = ecn_0B(\tau)$ is the peak current at τ, I_A is the Alfvén current and

$$U(s,\tau) = B(s)R_{56}(s) - B(\tau)R_{56}(\tau), \tag{5}$$

$$V(s,\tau) = B(s)R_{51}(s) - B(\tau)R_{51}(\tau), \tag{6}$$

$$W(s,\tau) = B(s)R_{52}(s) - B(\tau)R_{52}(\tau). \tag{7}$$

An approximated analytical solution for the bunching at the end of the chicane can be found by iteration, neglecting the microbunching induced by energy modulation in the same dipole, i.e. considering staged amplification from one dipole to another. The problem reduces to the determination of the following terms

$$\begin{aligned} b[k(s_3);s_3] = b_0[k(s_3);s_3] + \int_0^{L_b} ds_1 K(s_1,s_3) b[k(s_1);s_1] + \int_0^{2L_b} ds_2 K(s_2,s_3) b[k(s_2);s_2] + \\ + \int_0^{2L_b} ds_2 K(s_2,s_3)\int_0^{L_b} ds_1 K(s_1,s_2) b[k(s_1);s_1] \end{aligned} \tag{8}$$

where s_j is the coordinate inside the jth dipole. Transfer functions are

$$
\begin{aligned}
&R_{51}(s_1)=\frac{s_1}{\rho_0}, \quad R_{52}(s_1)=\frac{s_1^2}{2\rho_0}, \quad R_{56}(s_1)=\frac{s_1^3}{6\rho_0^2},\\
&R_{51}(s_2)=\frac{L_b-s_2}{\rho_0}, \quad R_{52}(s_2)\approx\frac{\Delta L s_2}{\rho_0}, \quad R_{56}(s2)\approx-\frac{\Delta L L_b}{\rho^2}s_2,\\
&R_{51}(s_3)=-\frac{L_b-s_3}{\rho_0}, \quad R_{52}(s_3)\approx\frac{2\Delta L L_b(s_3-L_b)}{\rho_0}, \quad R_{56}(s_3)\approx-\frac{2\Delta L L_b^2}{\rho^2}\equiv R_{56}\\
&R_{56}(s_1\to s_2)\approx-\frac{\Delta L}{\rho^2}(L_b-s_1)s_2, \quad R_{56}(s_2\to s_3)\approx-\frac{\Delta L}{\rho^2}(2L_b-s_2)s_3,\\
&R_{56}(s_1\to s_3)\approx-\frac{2\Delta L}{\rho^2}[(L_b-s_1)L_b+s_1 s_3].
\end{aligned}
\tag{9}
$$

We recall that, in absence of coherent synchrotron radiation (CSR), the bunching degradation is

$$
b_0[k(s);s]=b_0[k_0;0]e^{-k^2(s)R_{56}^2(s)\sigma_\delta^2(s)/2}\exp\Big[-\frac{k^2(s)\epsilon_0\beta_0}{2}\Big(R_{51}(s)-\frac{\alpha_0}{\beta_0}R_{52}(s)\Big)^2-\frac{k^2(s)\epsilon_0}{2\beta_0}R_{52}^2(s)\Big] \tag{10}
$$

where we have included also the coordinate dependence of σ_δ.
The first term in equation (8) for $s_3=L_b$, hereafter denoted with subscript "f", becomes

$$
b_0[k_f;f]=\exp\Big(-\frac{\bar{\sigma}_\delta^2(f)}{2(1+hR_{56})^2}\Big)b_0[k_0;0] \quad \text{with} \quad \bar{\sigma}_\delta^2(f)=k_0R_{56}(\sigma_\delta^2(0)+AL_b). \tag{11}
$$

Indeed, this is the only situation in which the IBS has an impact on the microbunching gain. This consideration is true for both initial density and energy modulation.
The contribution from the first dipole, considering that the dominant part of the damping is due to the term proportional to $R_{51}(s_1)$ in equation (10), is

$$
\int_0^{L_b}ds_1K(s_1,s_3)b[k(s_1);s_1]=\frac{\sqrt{\pi}A\bar{I}_f}{2\bar{\sigma}_x}\exp\Big(-\frac{\bar{\sigma}_\delta^2(0)}{2(1+hR_{56})^2}+D^2\Big)\Big[erf\Big(\bar{\sigma}_x+D\Big)-erf\Big(D\Big)\Big]b_0[k_0;0] \tag{12}
$$

where... and we have introduced the new quantity D

$$
D=\frac{L_bk_0^2R_{56}^2B(f)^2A}{2\bar{\sigma}_x}. \tag{13}
$$

D quantifies the contribution of growing energy spread. The possibility to ignore IBS effect translate to $D\ll 1$, or

$$
\lambda_0\gg\lambda_{crit}=\frac{\pi L_bR_{56}^2B(f)^2A}{2\sqrt{\epsilon_0\beta_0}\nu} \tag{14}
$$

where ν is the chicane bending angle.
We now recall the $H(t)$ function and rewrite it with the inclusion of terms describing the IBS effect. The evaluation of the modified $H(t)$ allows us to quantify the relevance of those terms to the overall dynamics.

$$
H(t)=\exp\Big[-\bar{\sigma}_x^2\frac{(1-2t+\alpha_0\phi t)^2+\phi^2t^2}{(1+hR_{56}t)^2}-\frac{\bar{\sigma}_\delta(t)}{2(1+hR_{56}t)^2}\Big(t^2+\frac{(1-t)^2}{(1+hR_{56})^2}\Big)\Big] \tag{15}
$$

in which $\phi=2\Delta L/\beta_0$.
IBS contributes with terms at first, second and third order in t in the numerator. We keep only the first two, accordingly to the assumptions of the model. The expression for the added contributions is

$$
\exp\Big[\frac{L_bk_0^2R_{56}^2B(f)^2A}{2(1+hR_{56}t)^2}t(1-2t)\Big]=\exp\Big[\frac{\bar{\sigma}_xD}{\pi(1+hR_{56}t)^2}t(1-2t)\Big] \tag{16}
$$

and we therefore find that IBS is negligible when:

$$|L_b R_{56}^2 B(f)^2 A| \ll |4\epsilon\beta\theta_{chicane}^2(\alpha_0\phi - 2)| \quad \text{and} \quad |L_b C^2 R_{56}^2 A| \ll |\epsilon\beta\theta_{chicane}^2[4(\alpha_0\phi-2)^2+\phi^2]|, \tag{17}$$

for the linear and quadratic term respectively. However, the first condition is sufficient since it implies also the second one. So, rewritten in terms of λ_{crit}, the first condition becomes

$$\bar{\sigma}_x\lambda_0 \gg \frac{\lambda_{crit}}{2|\alpha_0\phi - 2|}. \tag{18}$$

Again, condition (14) is stronger than condition (18), demonstrating the statement in the paper. For instance, at the FERMI first magnetic bunch compressor (BC1)[3], the value of λ_{crit} is in a range between 10^{-8} and 10^{-9}. Therefore, since the FEL process is affected by modulations at the micron scale, we are legitimized to neglect all the contributions.

References

1. Tsai, C.Y. et al. Theoretical formulation of phase space microbunching instability in the presence of intrabeam scattering for single-pass or recirculation accelerators. *Phys. Rev. Accel. Beams* **23**, 124401 (2020).
2. Huang, Z., Kim, K. J. Formulas for coherent synchrotron radiation microbunching in a bunch compressor chicane. *Phys. Rev. Spec. Top. Accel. Beams* **5**, 074401 (2002).
3. Brynes, A.D., et al. Characterisation of microbunching instability with 2D Fourier analysis. *Sci Rep* **10**, 5059 (2020).
